# Effect of collaborative quality improvement on stillbirths, neonatal mortality and newborn care practices in hospitals of Telangana and Andhra Pradesh, India: evidence from a quasi-experimental mixed-methods study

**DOI:** 10.1186/s13012-020-01058-z

**Published:** 2021-01-07

**Authors:** Karen Zamboni, Samiksha Singh, Mukta Tyagi, Zelee Hill, Claudia Hanson, Joanna Schellenberg

**Affiliations:** 1 Department of Disease Control, London School of Hygiene and Tropical Medicine, Keppel Street, London, WC1E 7HT UK; 2 Public Health Foundation, India, Kavuri Hills, Madhapur, Hyderabad, India; 3 Institute for Global Health, University College London, London, UK; 4 Department of Public Health Sciences, Karolinska Institutet, Stockholm, Sweden

**Keywords:** Quality improvement, Evidence-based practices, Neonatal mortality, Newborn care, India, Sick newborn babies

## Abstract

**Background:**

Improving quality of care is a key priority to reduce neonatal mortality and stillbirths. The Safe Care, Saving Lives programme aimed to improve care in newborn care units and labour wards of 60 public and private hospitals in Telangana and Andhra Pradesh, India, using a collaborative quality improvement approach. Our external evaluation of this programme aimed to evaluate programme effects on implementation of maternal and newborn care practices, and impact on stillbirths, 7- and 28-day neonatal mortality rate in labour wards and neonatal care units. We also aimed to evaluate programme implementation and mechanisms of change.

**Methods:**

We used a quasi-experimental plausibility design with a nested process evaluation. We evaluated effects on stillbirths, mortality and secondary outcomes relating to adherence to 20 evidence-based intrapartum and newborn care practices, comparing survey data from 29 hospitals receiving the intervention to 31 hospitals expected to receive the intervention later, using a difference-in-difference analysis. We analysed programme implementation data and conducted 42 semi-structured interviews in four case studies to describe implementation and address four theory-driven questions to explain the quantitative results.

**Results:**

Only 7 of the 29 intervention hospitals were engaged in the intervention for its entire duration. There was no evidence of an effect of the intervention on stillbirths [DiD − 1.3 percentage points, 95% CI − 2.6–0.1], on neonatal mortality at age 7 days [DiD − 1.6, 95% CI − 9–6.2] or 28 days [DiD − 3.0, 95% CI − 12.9—6.9] or on adherence to target evidence-based intrapartum and newborn care practices. The process evaluation identified challenges in engaging leaders; challenges in developing capacity for quality improvement; and challenges in activating mechanisms of change at the unit level, rather than for a few individuals, and in sustaining these through the creation of new social norms.

**Conclusion:**

Despite careful planning and substantial resources, the intervention was not feasible for implementation on a large scale. Greater focus is required on strategies to engage leadership. Quality improvement may need to be accompanied by clinical training. Further research is also needed on quality improvement using a health systems perspective.

**Supplementary Information:**

The online version contains supplementary material available at 10.1186/s13012-020-01058-z.

Contributions to the literature

- Quality improvement collaboratives are a widely used approach, but evidence of their effectiveness is mixed. We conducted an evaluation of a quality improvement collaborative aiming to reduce newborn mortality and stillbirths, targeting labour rooms and newborn care units of 60 hospitals in two Indian states.
- We found no evidence that the intervention reduced stillbirths or neonatal mortality, nor that it improved targeted intrapartum and newborn care practices in labour rooms and newborn care units.
- Implementation of the intervention was challenging, and there was high attrition from participating hospitals.
- This study contributes to an emerging body of evidence suggesting caution in considering quality improvement collaboratives an effective short-term intervention. Much attention is needed on engaging leadership and building capacity to enable quality improvement at scale.

## Introduction

**Fig. 1:**
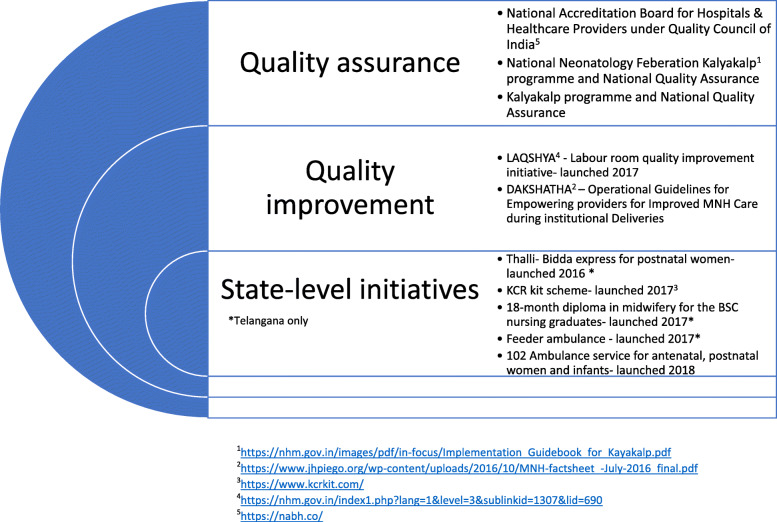
Context panel. Maternal and newborn health initiatives affecting labour room and newborn care units in Andhra Pradesh and Telangana

Globally, poor quality care contributes to over 1 million newborn deaths each year [1]. India is a high-burden country, with around 760,000 yearly newborn deaths, and an estimated neonatal mortality rate of 24 deaths per 1000 live births, with variations across states, wealth quintiles and urban-rural settings [2]. In the last decade, the Indian government has invested heavily in demand-side programmes, which resulted in improvements in institutional deliveries and skilled birth attendance [3]. In line with the Indian Every Newborn Action Plan [4], four levels of neonatal care have been established: Newborn Care Corners at all places offering childbirth care, providing essential care at birth and newborn resuscitation; Level I Newborn Stabilisation Units providing management of low birthweight babies not requiring intensive care and stabilisation of sick newborns before referral; Level II Special Newborn Care Units at district and subdistrict hospitals, providing care to sick newborns except ventilation and surgery; and Level III Neonatal Intensive Care Units [5]. Considerable progress has been made in operationalising these structures through standardised infrastructure guidelines, human resource standards and a system for reporting data on facility-based newborn care [5, 6]. However, quality in newborn care remains suboptimal due to limited adherence to care protocols, a weak referral system and admission overload [5, 7–9]. National quality improvement initiatives and quality assurance schemes, such as that of the National Neonatology Federation, have recently been introduced (see Fig. 1). A nationwide quality of care network has been established, spreading the adoption of quality improvement (QI) strategies [10].

The Safe Care, Saving Lives programme (SCSL), implemented by ACCESS Health International (ACCESS), an international NGO, used a collaborative quality improvement approach, adapted from the Institute of Healthcare Improvement [11] to reduce neonatal mortality. In this approach, teams from multiple hospitals work together to improve implementation of evidence-based practices (EBPs), in this case EBPs for intrapartum and newborn care. Twenty EBPs were identified by neonatologists and obstetricians, addressing the three main drivers of neonatal survival through: (1) neonatal sepsis prevention and management, (2) prevention and management of complications from prematurity and (3) reliable intrapartum care and newborn resuscitation [12]. Teams were supported by quality improvement coaches to use rapid cycle tests of change to achieve a given improvement aim and attend “learning sessions” to share improvement ideas, experience and data on performance [11]. Quality improvement collaboratives (QICs) are a widely used approach. Collaboration between teams can shorten the time required to identify challenges to EBP implementation and can provide an external stimulus for innovative problem-solving [13]. Evidence on QICs effectiveness is mixed [14, 15] and of variable quality [14, 16], but recent robust studies reported positive results for newborn health outcomes [17]. SCSL developed a collaborative of all hospitals empanelled into a government-sponsored health insurance scheme covering care for severely sick newborns: the Aarogyasri Health Care Trust [18] and the Dr Nandamuri Taraka Rama Rao Vaidya Seva in Telangana and Andhra Pradesh, respectively. The schemes provide the poor with access to secondary and tertiary newborn care in both private and public facilities. SCSL targeted Level II Special Newborn Care Units and Level III Neonatal Intensive Care Units, which we refer to together as “newborn care units” (NCUs), and labour wards in 60 public and private hospitals in Telangana and Andhra Pradesh.

We conducted an external mixed-methods evaluation of the SCSL programme. Here we report on the following: (i) effects on the implementation of essential evidence-based maternal and newborn care practices; (ii) the impact on the stillbirth rate and neonatal mortality rate in labour wards and neonatal care units; (iii) programme implementation including challenges and adaptations to the context and (iv) observed mechanisms of change and their relationship to contextual factors.

## Methods

### Study design, allocation and setting

We used a quasi-experimental plausibility design with a nested process evaluation, details of which are presented elsewhere [12]. The intervention targeted all 85 hospitals that were empanelled in the health insurance schemes, through a phased intervention roll-out organised in three waves (see Table 1). Wave 1, where the intervention was piloted and refined [12], involved 25 hospitals that volunteered to participate after a programme launch. These were excluded from our study. The 60 remaining hospitals represented the study sample. The allocation of the 60 eligible hospitals to waves 2 and 3 was initially planned using randomisation. However, before implementation, ACCESS purposely reallocated 5 facilities to enable collaboration between hospitals in the same newborn referral cluster and relative geographical proximity. This created a non-randomised, quasi-experimental study. In the study sample, 29 hospitals received the intervention in wave 2 between April 2017 and July 2018, and 31 represented the comparison group, where wave 3 roll out was planned from July 2018. However, the wave 3 group did not receive the intervention because permission for the programme was withdrawn in Andhra Pradesh in late 2017, and a programme review by the donor recommended that ACCESS intensified support to waves 1 and 2 hospitals, instead of expanding into new sites. Hospital characteristics are reported in Table 2.

**Table 1:**
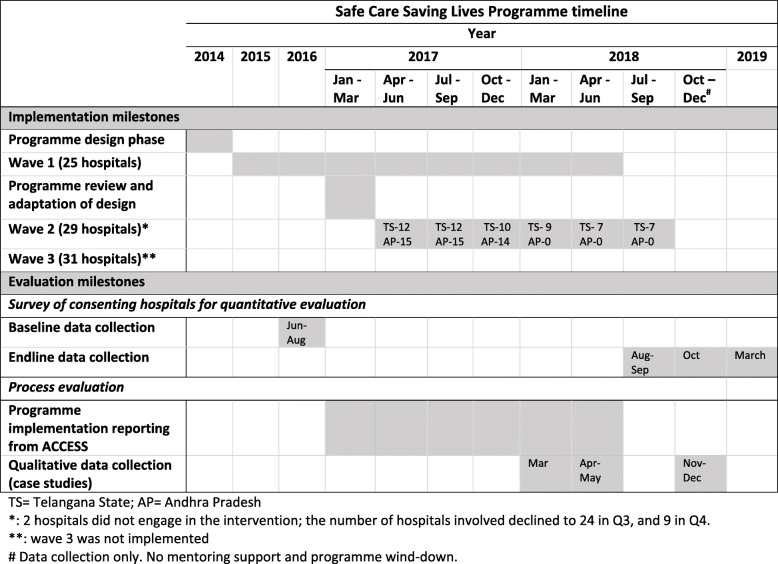
Implementation and evaluation timeline

**Table 2:** Infrastructure and human resources in included hospitals

	Baseline		Endline	
	Intervention	Comparison	Intervention	Comparison
Agreed participation in baseline and endline assessment	**25**	**27**	18	21
**Facility assessment done in labour room**	**20**	**19**	14	17
Public secondary/college	15/1	11/3	12/1	11/2
Private secondary/college	1/3	2/3	1/1	1/2
Mean no. of deliveries per month	369	171	459	317
Median (IQR)	315 (157–500)	166 (32–247)	426 (253–636)	233 (85–516)
Mean no. beds	8	6	6	4
Median (IQR)	5 (3–11)	2 (2–10)	4 (3–8)	3 (2–7)
Hospital has an operating theatre	18 (90%)	16 (84%)	13 (87%)	13 (81%)
Mean no. of obstetricians per 10 beds	4	9	10	10
Median (IQR)	3 (2–7)	5 (3–10)	5 (3–8)	8 (5–15)
**Facility assessment done in NCU**	**24**	**25**	15	20
Public secondary/college	15/2	10/3	11/1	11/2
Private secondary/college	4/3	10/2	3/0	5/2
Have a breastfeeding room*	21 (88%)	20 (80%)	11 (85%)	14 (82%)
Have a Kangaroo Mother Care room	16 (67%)	10 (40%)	11 (79%)	13 (72%)
Mean no. of admission per month	77	72	91	69
Median (IQR)	67 (28–86)	51 (28–106)	65 (53–153)	53 (18–96)
Mean no. beds in NCU	19	16	19	20
Median (IQR)	18 (14–20)	16 (10–20)	20 (12–20)	20 (13–22)
Mean monthly admission to bed ratio	5	4	5	4
Median (IQR)	4 (3–5)	4 (2–6)	4 (3–8)	3 (1–6)
Mean no. of paediatricians	4	4	3	3
Median (IQR)	3 (2–7)	3 (1–6)	2 (1–4)	3 (1–5)
Mean no. of nurses	5	4	9	9
Median (IQR)	5 (3–6)	4 (2–5)	7 (6–14)	8 (3–13)
Mean no. of paediatricians per 10 beds	2	3	1	2
Median (IQR)	2 (1–3)	2 (1–3)	1 (0–3)	2 (1–3)
Mean no. of nurses per 10 beds	6	8	5	5
Median (IQR)	6 (3–8)	7 (5–8)	5 (4–7)	5 (3–7)

Note: *5 missing at endline (2 intervention, 3 comparison)

For the qualitative component, we used a two-round multiple case study design to evaluate intervention adaptation, contextual factors, and mechanisms of change. We purposely selected four case study hospitals in Telangana. We aimed to include a private and public hospital and a medical college and to balance high and medium admission caseloads, hypothesising that these characteristics would influence their engagement in the programme.

### Participants

The study site was two Indian states of Telangana and Andhra Pradesh, which have a slightly better socio-economic situation than India’s average [12]. The 60 participating hospitals included 28 public secondary hospitals, 6 public medical colleges, 20 private tertiary hospitals and 6 private medical colleges with high neonatal mortality rates as described elsewhere [19]. We included women seeking childbirth care and neonates admitted to NCUs.

### Intervention

**Fig. 2:**
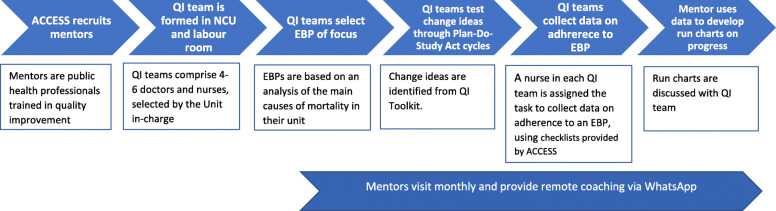
Implementation strategy outline for Safe Care, Saving Lives

Figure 2 summarises intervention implementation, described elsewhere in detail [12]. To evaluate intervention delivery, we used quarterly programme data reported by ACCESS on EBP implementation in each hospital, reported under programme implementation.

ACCESS also planned to facilitate learning sessions among participating hospitals. However, only one mini-collaborative was set up which, upon request of participating hospitals, focused on newborn referral pathways instead of EBPs. Therefore, this component was not included in the evaluation.

### Outcomes

Primary outcomes were as follows: (1) the stillbirth rate, defined as number of foetuses born without any signs of life and weighing 1000 g or more, as a proportion of all births; (2) 7-day and (3) 28-day neonatal mortality rate after admission to a neonatal care unit, defined as babies who died before they completed 7 or 28 days of life, as a proportion of all babies admitted to the neonatal care unit. Deaths post-discharge but before 7 or 28 days of life were included. Secondary outcomes related to an improvement in the 20 intrapartum and newborn care practices targeted by the programme. Indicator definitions were mostly consistent with those used by the programme and aligned to international standards (Additional file 1).

### Sample size

We based our sample size on the 3 primary impact indicators of the stillbirth rate in the labour ward and the 7-day and 28-day neonatal mortality rate after admission to the newborn care unit. We used the formula proposed by Hayes and Moulton for unmatched clusters [20] and estimates of the k-factor, output and impact indicators from our baseline assessment. In each hospital, we aimed to include 260 observations from birth registers in the previous month and 190 phone interviews and newborn register data combined to be able to detect a 35% reduction of stillbirths and 20% reduction in mortality with 80% power [12].

### Quantitative data collection

Our baseline and endline surveys assessing primary and secondary outcomes were independent from the internal programme monitoring and included (i) labour room and newborn care unit readiness checklists, (ii) case note abstraction and observations of admissions and (iii) register abstraction in labour wards and newborn care units and (iv) face-to-face and telephonic interviews with mothers to estimate neonatal mortality after discharge from labour rooms and newborn care units [21]. We used android-based tablets (Lenovo) with an SQLite application with in-built skips and ranges to improve quality of data. Data were saved daily and uploaded on a safe server weekly.

Researchers from the Public Health Foundation of India (PHFI) collected data at baseline and endline over a period of 6 days per hospital. We employed six teams at baseline and three teams at endline, due to its smaller scope. To minimise inter-observer bias, one third of team members worked on both baseline and endline surveys.

Baseline data collection ran from June to August 2016. The majority of endline data collection was conducted from August to October 2018, after the programme end. However, due to delays in receiving permissions from the hospitals and suspension of PHFI’s license to receive foreign funding under the Foreign Contribution (Regulation) Act, data collection in 12 hospitals took place in March 2019 [22].

### Statistical analysis

Data were clustered at hospital level, so we computed cluster (hospital) summary estimates and tabulated primary and secondary outcome indicators, by intervention and comparison groups. We used a difference-in-difference (DiD) approach to assess the effect of the intervention on primary and secondary outcomes [23] using Stata version 15.1. In view of major investments in maternal and newborn care in the two states over the course of this study, we also conducted a post hoc analysis of all indicators in the study population to describe changes in primary and secondary outcomes over time.

### Case study design, data collection and analysis

**Fig. 3:**
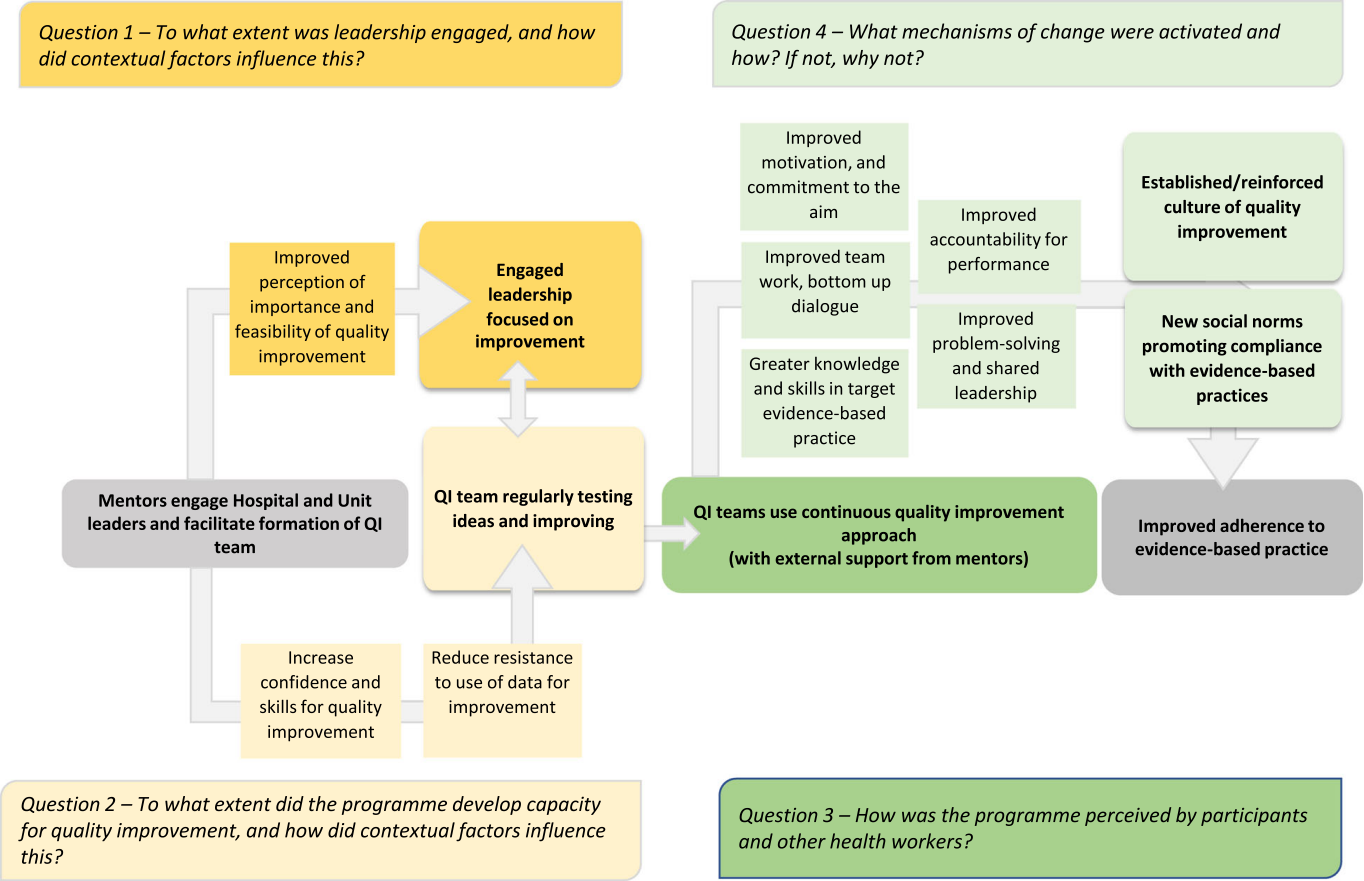
Programme theory

For the nested qualitative study, we first developed a theory of change through a participatory workshop with programme implementers [12], then refined it integrating relevant theory, informed by a systematic review [24, 25]. We developed four theory-based questions for the enquiry of context and mechanisms of change (Fig. 3) and conducted semi-structured interviews to explore participants’ understanding of the intervention, their perception of the priorities, barriers and enablers to newborn care quality improvement and their views of positive and negative changes occurring in their units. In the four case studies, we interviewed hospital leaders, 4–5 QI team members and ACCESS mentors. We drew the sample purposively from a list provided by ACCESS, balancing seniority and cadres. Interviews were conducted in English or Telugu after translation, back-translation and piloting of interview guides, and undertaken in two rounds in March–April and November 2018. In round two, we also interviewed 1–2 health workers not involved in the QI teams to understand the changes occurring in the unit and explore sustainability. In the three public hospitals, we completed 11–13 interviews, while in the private hospital we conducted 5 interviews in the NCU only. Overall, we conducted 31 interviews in round 1 and 11 in round 2.

Data quality assurance included (i) debriefing after each interview and on a weekly basis, (ii) production and review of transcripts while in the field or shortly after and (iii) discussion of a draft case study summary ahead of the final interview with the facility mentor. We used thematic content analysis using NViVO 12.1 based on a preliminary coding framework for the broad domains of implementation, context and mechanisms of change. Two researchers independently coded data using a deductive-inductive approach. We first applied the coding framework to the data and gradually refined it through discussion as interviews were coded. A final coding framework was agreed by both researchers. We completed analysis of single case studies first, then contrasted and synthesised key themes across case studies, to answer the theory-driven questions [26].

### Ethics

Ethical approval was granted from LSHTM (LSHTM Ethics Ref 10358) and PHFI’s Institutional Ethics Committee (IIPHH/TRCIEC/064/2015). Consent was obtained from each participating hospital prior to starting data collection and from each participant health worker and mother, after reading out an information sheet. Participants could withdraw or request to stop recording interviews at any time. Confidentiality was assured, as per institutional guidelines of research institutions.

## Results

### Programme implementation

**Fig. 4:**
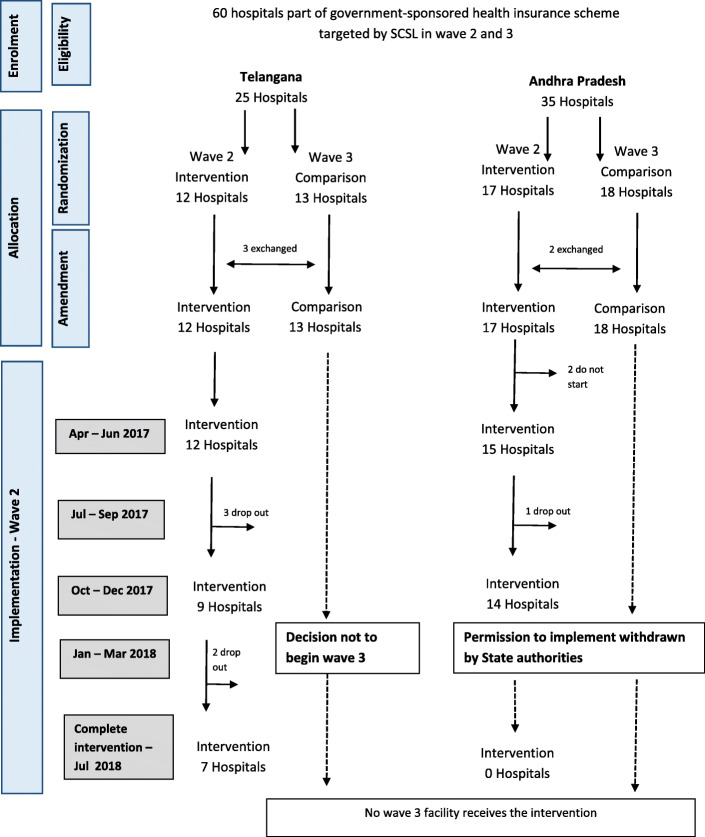
Intervention implementation flowchart.

Only 9 of the 29 hospitals recruited in wave 2 continued implementation for 12 months, and only 7 for 16 months (Fig. 4). Although the intervention promoted 20 EBP, a subset of 14 was implemented by any of these 9 hospitals (a mean of 5 per hospital), the commonest being hand hygiene, kangaroo mother care and anti-septic non-touch technique in NCUs and early breastfeeding in labour rooms (Table 3). Most EBP involving clinical protocol implementation were not adopted by any of the hospitals (Table 4).

**Table 3:** Implementation of evidence-based practices in wave 2 facilities (Telangana state only)

Hospital N.	Type	Level	No. EBPs implemented^**#**^	Sepsis				Prematurity					Birth Asphyxia				
				Labour rom		NCU		Labour room		NCU			Labour room				NCU
				Six cleans	Hand hygiene in vaginal exam	ANTT	Hand hygiene in NCU	Early BF	ANCS	TMA	KMC	Exclusive BF	High risk categorisation	NRP in high risk delivery	Parto-graph	Pre-delivery checklist	CPAP
1*	Public	Secondary	6	x	X	x	x	x			x						
2	Public	Medical college	9					x	x	x	x	x	x	x		x	x
3	Public	Secondary	5				x		x	x	x		x				
4	Public	Secondary	5			x	x	x			x	x					
5	Public	Secondary	8		X	x	x	x			x	x	x		x		
6	Public	Secondary	8			x	x	x	x	x	x		x	x			
7	Private	Secondary	2			x	x										
8*	Private	Secondary	5			x	x				x	x			x		
9*	Private	Secondary	1				x										
**Number of hospitals implementing EBP**				**1**	**2**	**6**	**8**	**5**	**3**	**3**	**7**	**4**	**4**	**2**	**2**	**1**	**1**

Based on data provided by implementing partner to donor in July 2018

*ANTT* anti-septic non-touch technique for IV line insertion, *ANCS* ante-natal corticosteroid administration, *BF* breastfeeding, *High risk* high risk categorisation at admission, *KMC* Kangaroo Mother Care, *NCU* newborn care unit, *NRP* neonatal resuscitation trained personnel, *CPAP* continuous positive air pressure, *TMA* temperature monitoring at admission

^#^EBP implementation defined as EBP a hospital is working on at the time of report

*Facility for which we have no data due to non-consent to study

**Table 4:** Evidence-based practices not implemented

Setting	PBPs not implemented	No.
	**Sepsis package**	**4**
Labour room	Antibiotics for women at risk of sepsis	
NCU	Protocol for central vascular catheter	
NCU	Prevent ventilator associated pneumonia	
NCU	Antibiotics for neonates born to mothers with risk of sepsis	
	**Prematurity package**	**0**
	**Birth Asphyxia package**	**2**
Labour room	Compliance with oxytocin infusion protocol	
Labour room	Resuscitation with bag and mask	
	**Total**	**6**

### Outcome and impact results

**Fig. 5:**
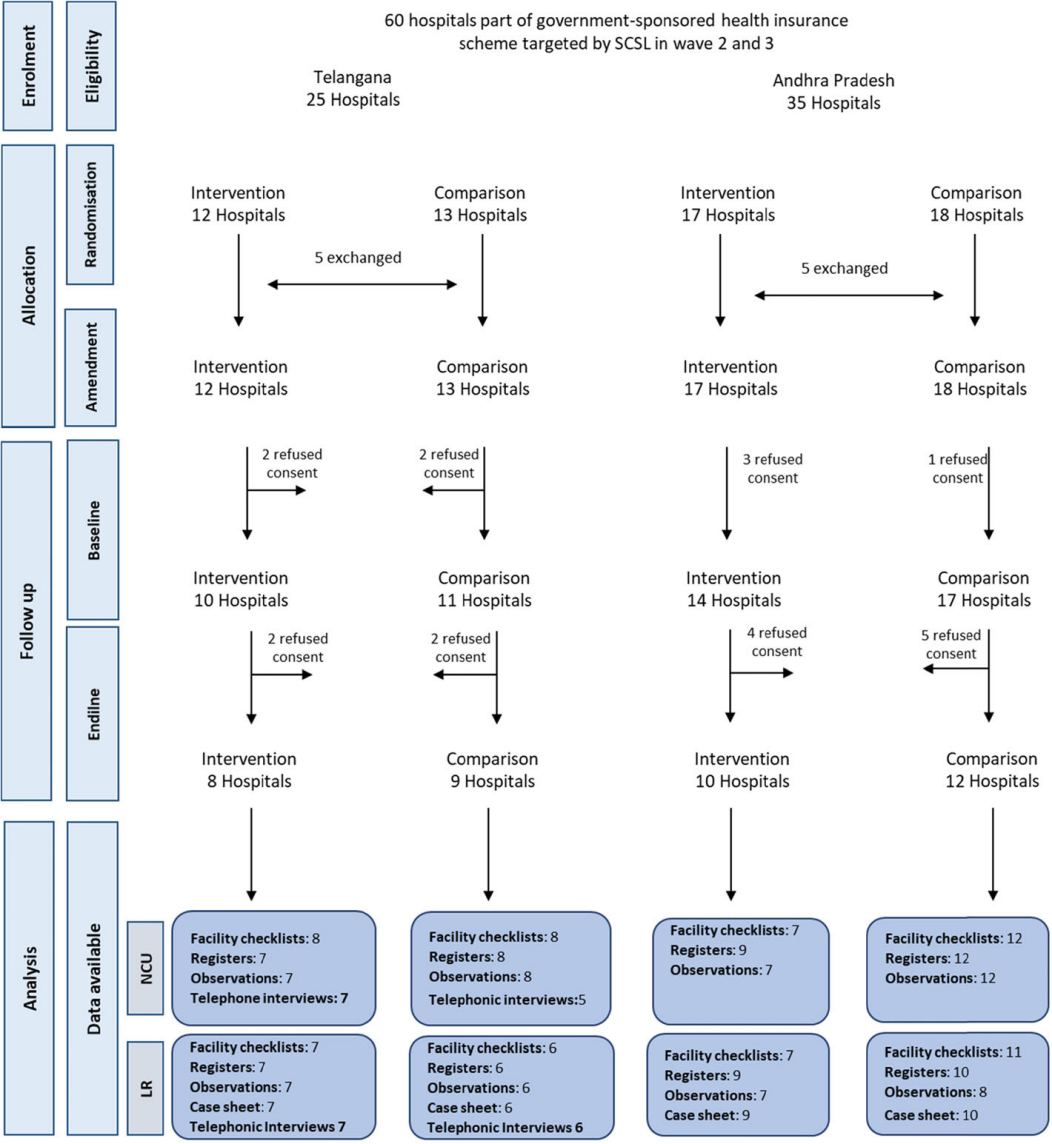
Study flowchart

Before-after data is available from 39 hospitals because 8 at baseline and further 13 at endline did not grant consent (Fig. 5). We completed 12,054 register abstractions in labour rooms and 1067 telephonic interviews at endline, a substantial increase from the 6466 and 866 completed at baseline respectively. At baseline, stillbirths represented 2.8% (95% CI 2.1–3.6) and 1.4% (95% CI 0.5–2.3) of hospital births in the intervention and comparison group, respectively. The 7-day and 28-day mortality rates were estimated at 4.9% (95% CI 1.1–8.8) and 7.6% (95% CI 1.8–13.5) of newborns admitted in NCUs in the intervention group and 6.0% (95% CI 1–11) and 8.0% (95% CI 0.8–15.1) in the comparison group respectively.

There was no evidence of an effect of the intervention on stillbirths [DiD − 1.3 percentage points, 95% CI − 2.6–0.1, *p* = 0.073]; on neonatal mortality at age 7 days [DiD − 1.6 percentage points, 95% CI − 9–6.2, *p* = 0.689] or 28 days [DiD − 3 percentage points, 95% CI − 12.9–6.9, *p* = 0.546], or on adherence to evidence-based practices (Table 5).

**Table 5:** Endline indicator summary

Indicator	Baseline		Endline		Difference in difference (DiD) effect (95% CI)	***p*** value of DiD
	Intervention ***N*** = 18	Comparison ***N*** = 21	Intervention ***N*** = 18	Comparison ***N*** = 21		
	Mean (95% CI)		Mean (95% CI)			
**Primary outcomes (impact indicators)**						
1. % of stillbirth of all hospital deliveries	2.8 (2.1–3.6)	1.4 (0.5–2.3)	0.9 (0.4–1.4)	0.5 (0.2–0.9)	− 1.3 (− 2.6–0.1)	0.073
2. % of neonates dying before the age of 7 days among those admitted to the newborn care unit	4.9 (1.1–8.8)	6.0 (1–11)	1.2 (0.1–2.4)	0.5 (− 0.5–1.5)	− 1.6 (− 9–6.2)	0.689
3. % of neonates dying before the age of 28 days among those admitted to the newborn care unit	7.6 (1.8–13.5)	8.0 (0.8–15.1)	1.4 (0.1–2.6)	1.7 (− 0.7–4.3)	− 3.0 (− 12.9–6.9)	0.546
**Secondary outcomes (EBP indicators)**						
**Delivery care practices in labour rooms**						
1. Percentage of high-risk assessments correctly flagged	37 (27–46)	35 (23–48)	19 (12–27)	20 (8–31)	− 2 (− 23–19)	0.841
2. Percentage of admissions where essential information was documented in partograph and attached to case notes	8 (0–16)	10 (0–23)	17 (8–26)	13 (3–22)	6 (− 14–26)	0.545
3. Percentage of admissions where safe childbirth checklist used and attached to case notes	12 (1–26)	9 (0–21)	53 (31–75)	29 (10–49)	21 (− 14–55)	0.232
4. Percentage of vaginal examinations where hygiene standards are met	17 (− 6–39)	29 (0–66)	20 (3–37)	17 (1–33)	15 (− 29–59)	0.495
5. Percentage of deliveries where the six cleans were adhered to	0 (− 0.4–1.3)	5 (0–15)	11 (0.2–22)	8 (0–26)	8 (− 11–26)	0.419
**Newborn care practices in Newborn Care Units**						
6. Percentage of babies seen in the neonatal care admission ward for whom temperature was measured within 15 min	49 (24–73)	52 (23–81)	22 (0–44)	51 (33–70)	− 26 (− 76–23)	0.285
7. Percentage of patient contacts where hygiene standards are met	6 (1–12)	7 (2–13)	49 (34–65)	38 (22–53)	12 (− 11–36)	0.292
8. Percentage of cannulations where hygiene standards are met	13 (0–32)	7 (0–21)	32 (9–54)	18 (1–35)	7 (− 29–44)	0.692
9. Percentage of babies discharged from newborn care unit who were exclusively breastfed at first interview after discharge	93 (86–99)	94 (89–99)	70 (59–82)	73 (56–89)	− 1 (− 19–16)	0.870
10. Percentage of mothers in SNCU that reported being assisted for kangaroo mother care	22 (5–39)	39 (22–57)	59 (40–78)	56 (38–63)	20 (− 15–56)	0.260

The post hoc analysis of changes in primary and secondary outcomes over time indicated marked improvements in both implementation and comparison groups combined in: stillbirths [from 1.9 to 0.7%, − 1.2 percentage points, 95%CI − 1.8 to − 0.7, *p* < 0.001]; 7-day mortality [from 5.4 to 0.9%, − 4.5 percentage points, 95%CI − 7.6 to − 1.4, *p* = 0.009]; 28-day mortality [from 7.7 to 1.5%, − 6.2 percentage points, 95% CI − 10.3 to − 2.1, *p* = 0.007). A few target EBPs also improved in both groups combined: hand hygiene in NCUs [from 6 to 43%, 37 percentage points, 95% CI 25–48, *p* < 0.001]; use of safe birth checklists in labour room [from 11 to 41%, 30 percentage points, 95% CI 14–47, *p* = 0.0008]; and assistance for kangaroo mother care in NCUs [from 34 to 58%, 24 percentage points, 95% CI 3–45, *p* = 0.0257]. There was no evidence that this increase was stronger in the intervention compared to the comparison group (Table 5), and no evidence of a change in the other secondary outcomes (see Additional file 2).

### Case study (CS) analysis

This section presents findings against the 4 theory-driven process evaluation questions outlined in Fig. 3. Table 6 describes the case study setting and implementation. Additional file 3 provides detailed qualitative results.

**Table 6:** Case study characteristics and programme implementation details

	Case study 1	Case study 2	Case study 3	Case study 4
**Key characteristics**				
**Type and level**	Public—medical college**	Private—secondary	Public—medical college**	Public—secondary
**Area**	Urban	Urban	Urban	Rural/tribal
**Monthly admissions to NCU**	103	59	152	145
**No. beds in NCU**	11	18	18	Missing
**Paediatricians per 10 beds in NCU** [state average 2]	2.7	1.7	1.1	n/a
**Nurse per 10 bed in NCU** [state average 7]	11.8	6.7	6.1	n/a
**Monthly deliveries**	1153	n/a	375	325
**Baseline performance in NCU (selected indicators)**				
**% of occasions when hand hygiene was followed in NCU** [state average 16%]	15	42	0	0
**% observed babies on exclusive breastfeeding** [state average 72%]	91	100	96	75
**Implementation**				
**Duration of implementation (months)**	April 2017 January 2018 (10)	June–December 2017 (6)	July 2017 July 2018 (13)	July 2017 July 2018 (13)
**Total no. EBPs at programme end (based on interview)**	2	0	6	9
**Total no. EBP**^ **(based on programme reports)**	5	2	5	8
**NCU**				
**QI team**	Active	No QI team	Active	Active
**Focus EBP (based on participants’ interviews)**	Hand hygiene ANTT	0	Hand-hygiene TMA KMC	Hand hygiene ANTT Exclusive BF KMC TMA
**Labour room**				
**QI team**	Not formed	No labour room	Formed but unstructured QI work	Active
**Focus EBP (based on participants’ interviews)**	None	n/a	ANCS HRC [Vitamin K administration]	NRP at delivery HRC Early BF ANCS

*ANTT* anti-septic non-touch technique for IV line insertion, *ANCS* ante-natal corticosteroid administration, *BF* breastfeeding, *HRC* high risk categorisation at admission, *KMC* Kangaroo Mother Care, *NCU* newborn care unit, *NRP* neonatal resuscitation trained personnel, *TMA* temperature monitoring at admission

^#^At baseline

^*^These were public secondary facilities at baselines, then accredited as medical colleges while the programme was ongoing

^Discrepancies are as follows:

- Case study 1: qualitative interviews did not confirm QI activities on 3 practices in the labour room. Participants referred to additional practices, but suggested they had been working on these before this programme and were supported by concurrent programmes

- Case study 2: programme reports include practices for which the facility provided monthly data; however, use of the QI approach was not confirmed by qualitative interviews

- Case study 3: vitamin K was not in the SCSL change package. It was introduced in LR to rationalise over-admission in NCU where the only reason for referral to NCU was vitamin K administration

- Case study 4: interview participants also referred to exclusive breastfeeding, for which the facility did not collect data

#### To what extent was leadership engaged, and how did contextual factors influence this?

Participants saw leaders’ role as essential to champion and model new behaviour and to provide administrative support and resources. The case studies offered mixed views about the extent to which leadership had the skills to motivate staff, were engaged in the initiative and were driving new behaviours. Three key contextual challenges to proactive leadership emerged. First, professional hierarchies and boundaries did not allow the creation of shared leadership across doctors and nurses, resulting in limited multi-professional collaboration. Second, top-down management styles hindered junior doctors’ active participation in quality improvement as they did not feel empowered to make suggestions to their superiors.

As an obstetrician… if new initiatives are to be followed… we have to change behavior of doctors and nurses. We don’t think about it; as junior subordinates we don’t give any suggestions. It will be good if they (seniors) will take suggestions from us… it will be good for patients. CS1_Medical Officer Labour Room

Third, leaders lacked higher level pressure to prioritise quality improvement, resulting in limited engagement.

Generally there will be a resistance because […] quality is not compulsion to any Government hospital and it is their choice to implement it or not. If the leadership wants it strongly then the staff obviously do it… but they do it forcibly. If the staff wants to develop their own unit, they do it. CS1_Mentor

#### To what extent did the programme develop capacity for quality improvement, and how did contextual factors influence this?

The case studies provided little evidence that the intervention developed capacity for QI in a sustainable way. Selection of focus EBP was mostly based on consultation with the Unit Manager in the NCU or labour room, based on a gap analysis conducted by the mentor. Practices were prioritised based on ease of implementation, as opposed to a team reflection on the gap analysis, for example prioritising practices that the hospital was already working on, such as kangaroo mother care. Functionality of QI teams varied across the case studies (Table 6). Implementation of PDSA cycles was unstructured and mostly limited to the *do* and *study* part of the cycle [11]. In three cases (CS1, CS3 and CS4), interviewees reported limited understanding of the change package, and that new initiatives were implemented based on mentors’ suggestions, while in the fourth case study (CS2) respondents were not clear about the concept of testing ideas for improvement. The limited understanding of the QI approach is evidenced also by the discrepancy between what interviewees understood the EBP of focus to be and what emerged from process monitoring data (Table 6). Health workers involved in the QI interventions reported being tasked with collecting data for ACCESS to analyse, although they were largely unaware of the purpose of this exercise. In two cases (CS3 and CS4), interviewees reported discussing results with ACCESS, but not sharing findings with others in the unit. Contextual factors that challenged implementation and capacity building, according to respondents, included staff shortages and high staff turnover, perceptions of inadequate resources and resistance from staff due to low motivation and limited focus on outcomes.

I: Did she (mentor) discuss anything about improvement?

R: No she did not because there is no staff. […] Most of us are busy, whenever she visited us. You may have noticed it too. One sister has to look after 20 babies. It’s very difficult. CS3_Nurse NCU

It is difficult nobody wants to work. We take salaries and we don’t work. That is attitude of the people. Every sister wants to sit daily. CS4_NCU Manager

#### How was the programme perceived by participants and other health workers?

Participants did not engage in the programme in the way it was intended. In the private hospital and one medical college (CS1 and CS2), the intervention did not generate involvement beyond 1–2 committed individuals, and very few other interviewees were aware of the programme activities. The intervention appears to have been better received in the other two case study facilities, based on the detail with which implementation was explained and examples of change provided by respondents. In all case studies, the programme was perceived as an external assessment. Participants mostly described the process of quality improvement as compiling a checklist to audit compliance with a certain EBP and reporting to ACCESS.

R: They assess whether we are practicing hand wash or using hand rub. They observe us and if we are free, they come and also ask us.

I: What they do with assessment?

R: I think they tell unit-manager and medical officers

CS1_Nurse NCU

Participants directly involved in programme activities suggested that the programme increased their workload because of the burden of documentation.

I: Why has the use of the checklist stopped?

R: We are busy and there is nobody to ask about it. We monitor but not document. We guide each other orally

CS1_Round 2_ Nurse NCU

Respondents articulated other more pressing priorities for QI, for example increasing staff numbers. Also, they could not fully differentiate this intervention from other ongoing initiatives. Nevertheless, in the three public facilities, participants welcomed the training received (e.g. on handwashing), lamented the short-term duration of the programme, and suggested that further monitoring by ACCESS would have been welcomed to keep focus on EBPs.

#### What mechanisms of change were activated and how? If not, why not?

Given the challenges with implementation and the lack of an effect of the intervention, the analysis of mechanisms of change could not be conducted as intended. We report instead on the themes emerging from participants’ responses when asked about changes they were seeing in their practice, in their team or in their unit, recognising that these represent the view of a few highly involved staff rather than prevalent views in the target units. We also report on contextual challenges emerging from the case studies which may explain why these changes failed to involve the wider team and thus why the expected change did not occur.

In terms of positive changes, five themes emerged. First, interaction with mentors helped bring focus on the aim for improvement and new ideas. Second, participation in the intervention improved motivation and commitment to improving the target EBP. Interaction with mentors reinforced the importance of complying with the practice and helped expose gaps and challenge complacency and reframe the issue as a problem with a solution over which staff had control. Seeing results further reinforced motivation. Third, the intervention enhanced staff knowledge and capacity to perform a certain practice. Fourth, participation in the intervention increased the sense of personal responsibility of the QI champions involved, who saw themselves as leading change by example. Fifth, a few respondents conveyed that the intervention created a climate in which behavioural expectations, for example for handwashing, were clear, and where staff could challenge each other if they observed non-adherence to those behaviours.

Previously they used to not do that. But now after the quality improvement people have come they do compulsorily hand wash and use hand rub in between. If they forget also, we remind them. They don’t feel [bad] because we are seniors they know why we are saying. Now everyone is aware that they should do hands wash. CS4_Round 2_Staff Nurse NCU

However, only in case study 4 did it appear that these mechanisms were sustained to the end of the programme. In the others, as soon as external scrutiny from mentors waned, the use of QI tools was discontinued.

If you give us some work and ask us to do, we will perform that activity only if we know that you are going to come back tomorrow to verify the same. If you come once in a blue moon day and ask us to do something, then they will not do it. The staff needs to have fear that people are coming back to ask us again. CS3_Staff Nurse NCU

The change in a few individuals did not translate in a shared sense of responsibility for QI. Contextual factors mentioned above, including high workloads, team work regulated by professional hierarchies and top-down management styles, as well as limited systems for holding staff to account and rewarding performance, were mentioned as key challenges. Nevertheless, interviews in round two suggested that adherence to target practices that had received sustained effort, e.g. handwashing in NCUs, was continuing and was well-understood by all, even if monitoring of compliance had ended.

## Discussion

Our study adds robust and substantive evidence, combining impact and theory-driven process evaluation on a large-scale quality improvement programme in secondary and tertiary Indian hospitals [27–29].The intervention was not implemented as intended, and only 7 of the planned 60 hospitals implemented QI activities for 16 months: two thirds of the intervention group dropped out, and none of the comparison group started activities, contrary to the initial plans. We found no effect of the intervention on facility-based neonatal mortality and stillbirths, or on the adherence to evidence-based intrapartum and newborn care practices in labour rooms and newborn care units. However, we found evidence of improvements over time in both groups with regard to stillbirths, 7- and 28-day neonatal mortality, use of checklists at birth, assistance with kangaroo mother care and hand hygiene: it seems likely that these were due to other interventions.

We used a theory of change to understand how contextual factors influenced implementation and the hypothesised mechanisms of change. We found key bottlenecks to the pathways identified in the theory of change, namely challenges in engaging leaders and maintaining commitment; challenges in developing capacity for QI; and challenges in activating mechanisms of change at the unit level, rather than for a few individuals, and in sustaining these through the creation of new social norms for all target practices.

High attrition of participating hospitals reflects the challenge of sustaining institutional stakeholders’ buy-in in Andhra Pradesh, and of engaging hospital leaders, including hospital administrators and the Unit Incharge, particularly in private hospitals and medical colleges. The model for QIC was modified during implementation to respond to the challenge of generating and sustaining commitment. These included a fluid QI team, selection of EBPs based on feasibility rather than driven by the gap analysis, and an unstructured cycle for innovation testing, relying on external advice and data analysis, rather than facilitation of team reflection. As a result, the quality improvement approach was diluted and perceived mostly as a data collection and auditing exercise by some participants, as opposed to a bottom-up problem-solving opportunity. The lack of collaborative learning sessions, a key feature of the QIC approach, may have compounded the limited opportunity for QI capacity building, since the approach was extremely new for the context.

This evaluation supports the body of evidence emerging from rigorous studies of QIC which has mixed results [30–32] and suggests caution in concluding that QIC interventions are effective [15, 16]. In particular, our study is consistent with the findings of the most recent systematic review, which found that QICs are more effective in moderate and opposed to low-resource setting, and when combined with training [14]. In our study, staffing constraints severely impacted on health workers’ ability to engage in quality improvement. Although mentors delivered training on quality improvement and on non-clinical practices, e.g. hand washing techniques, the programme did not envisage training on new clinical practices, such as antenatal corticosteroid administration. Recent evaluations of QICs for newborn outcome improvement point to the importance of combining QI with problem analysis and clinical training [17]. The limited coherence between analysis of drivers of hospital mortality and selection of EBPs, the limited focus on EBPs requiring clinical practice changes and the emphasis on single EBPs as opposed to a whole change package of clinical and non-clinical interventions for the key driver of mortality in each hospital may partly explain the nil results.

A recent review on how and why QICs may improve outcomes highlights the need to contextualise QIC implementation and test mechanisms of change through greater use of theory in design and evaluation [24]. Our results confirm that QIC effectiveness is highly sensitive to context. Limited fidelity in application of PDSA approaches has been found in high-income settings as well [33], and high attrition is a common implementation challenge [34]. While some process evaluations have reported positive perceptions from participation in quality improvement [35], other studies have reported similar challenges in engaging leadership [34, 36]. This highlights the need to consider leadership engagement as part of the intervention, because this cannot be taken for granted. Therefore, QICs should not be considered a short-term intervention. The SCSL programme was initiated concurrently with other government initiatives, including quality assurance schemes, and was not, at least initially, aligned to these. This may explain the challenge of engaging hospital leaders. Our findings on leadership also suggest that in a context with strong professional hierarchies and boundaries, greater attention needs to be placed on ensuring that implementers have the professional credentials, the networks and status required to generate traction across all the health worker cadres whose behaviour is targeted. Developing strategies for leadership engagement may require greater understanding of health system factors and pressures and incentives for hospital leadership.

Similarly, building systems and skills for continuous use of data for decision-making and reducing resistance to this has been described elsewhere as “an intervention in itself*”,* requiring longer timeframes than expected [33]. Our findings are consistent with these. In our context, the challenges of building QI capacity were compounded by systemic constraints, such as high staff-patient ratios, high workloads and infrastructural challenges. This echoes the limitations of point of care interventions reported elsewhere [1, 9, 37–39], and that further attention to the enabling environment, or readiness for quality improvement, is necessary to improve intervention design and effectiveness [40].

Our qualitative results confirm the complexity of QIC interventions: more than a set of tools and approaches, QICs need to be designed and evaluated as social innovations, requiring change at multiple levels and adaptation to the context. Our qualitative analysis aimed to explore the cognitive, social and organisational changes brought about by participants’ engagement with the QIC intervention and how these could explain outcomes [28, 41, 42]. Our theory of change appears valid, as case studies confirmed most of the themes that had been hypothesised as mechanisms of change. In addition, case studies highlighted that the mentoring received brought new focus and new attention to a specific issue. This is consistent with quality improvement principles [13] and has been described elsewhere as a process of reframing [43]. In the theory of change, this could be conceptualised as the first key mechanism on the pathway to further changes (individuals need to perceive the severity and urgency of a problem in order to prioritise doing something about it) [44]. However, in our evaluation, we found that the changes reported by a few individuals involved in the intervention did not translate in a sustained shift to a culture of quality improvement at the level of units and hospitals, and there was no change in intended outcomes, therefore we cannot conclude that these acted as mechanisms of change. In addition to limited leadership engagement, this may have been due to three contextual factors: (i) staff workload and low motivation, preventing adequate implementation and active engagement in QI approaches; (ii) the challenge of mobilising a professionally diverse QI team for bottom-up gap analysis and discussion, due to professional boundaries and hierarchical processes for decision-making; and (iii) the prevailing working culture encouraging compliance to external requests, as opposed to self-reflection and problem-solving. At the root of our theory of change is normalisation process theory [25]. In line with this theory, our findings suggest that the contextual challenges did not enable participants to find the intervention coherent with their concerns, capabilities and priorities, resulting in limited ownership of the QI approach. This in turn hampered collective action and reflective monitoring [45]. Greater focus on organisational change mechanisms, as opposed to individual behaviour change, and on developing strategies that modify key contextual bottlenecks is necessary to improve intervention design [46, 47].

Our impact and outcome evaluation used a quasi-experimental design with externally assessed as opposed to self-reported outcomes, which is a strength. The integration of a rigorous process evaluation enables us to explain observed results. The theory-driven approach adds depth to our analysis and enables us to capture learning that is relevant to the wider debate on how to improve quality of maternal and newborn care, which is the major frontier for the achievement of universal health coverage.

We could not test whether outcomes relate to the intensity of the mentoring and coaching approach, due to challenge of capturing process data to define implementation strength reliably. Improved standardisation of process monitoring, for example standardising definitions on when an EBP is considered adopted may be useful in future evaluations of QIC. Because of the high attrition, we also lacked the statistical power to conduct such secondary analysis. We could not test mechanisms of change through a mediation analysis, due to the small sample in which the intervention was implemented. However, the qualitative work has contributed to a theory of change that may allow quantitative testing in future studies. Finally, groups could have differed in important ways at baseline because of lack of randomisation. However, implementation would have been even weaker had facilities been randomly allocated.

## Conclusion

Our evaluation of a QIC intervention in 60 secondary and tertiary hospitals with newborn care units in Telangana and Andhra Pradesh, India, found that the intervention did not improve adherence to target EBP or result in a measurable impact on neonatal mortality or stillbirths. Moreover, of the initial 29 hospitals intended to be included in the intervention, only 7 implemented the intervention for 16 months, suggesting that the intervention was not feasible in this context. The nested process evaluation highlights the need to consider the contextual challenge of engaging leaders: greater involvement of technical experts and alignment with national quality strategies may aid this. Building capacity for QI requires timely and consistent support. Using a theory of change can help to conceptualise individual and organisational changes and potential bottlenecks. Quality improvement may need to be accompanied by clinical training if target EBPs require changes in clinical practice. We highlight the need for further research on strategies for positioning quality improvement efforts within health systems, on quantitative testing of our QIC theory of change and on the optimal combination and intensity of training and QI.

## Supplementary Information

**Additional file 1:** Annex 1: List of indicators, data sources and number of observations.

**Additional file 2:** Annex 2: Post-hoc before and after comparison (intervention and comparison groups combined)

**Additional file 3:** Annex 3: Table 5 – Leadership, Table 6 – Contextual challenges for QI team mobilisation and capacity building, Table 7 - Perceptions of programme by health workers, Table 8 – mechanisms of change

## Data Availability

The datasets analysed during the current study are available in the LSHTM repository, Data Compass.

## Abbreviations

ACCESS: Access Health International
CS: Case study
DiD: Difference-in-difference
EBP: Evidence-based practice
NCU: Newborn care unit
QI: Quality improvement
QIC: Quality improvement collaborative
PDSA: Plan, Do, Study, Act
SCSL: Safe Care Saving Lives
