# Supplementary material for: Effect of collaborative quality improvement on stillbirths, neonatal mortality and newborn care practices in hospitals of Telangana and Andhra Pradesh, India: evidence from a quasi-experimental mixed-methods study
Source: Implement Sci. 2021 Jan 7;16:4. doi: 10.1186/s13012-020-01058-z (PMC7788546; doi:10.1186/s13012-020-01058-z)
Supplement: Supplementary file 1 — Additional file 1: Annex 1: List of indicators, data sources and number of observations. [file 13012_2020_1058_MOESM1_ESM.docx]

**ANNEX 1: List of indicators, data sources and number of observations.**

| **Indicator** | **Data source** | **N baseline** | | **N endline** | | |
| --- | --- | --- | --- | --- | --- | --- |
| **Outcome indicators** |  |  | |  | | |
| % of stillbirth of all hospital deliveries | Labour room register | 6466 | | 12054 | | |
| % of neonates dying before the age of 7-days among those admitted to the newborn care unit | Telephonic Interviews with mothers after discharge | 866 | | 1067 | | |
| % of neonates dying before the age of 28-days among those admitted to the newborn care unit |  |  |  |  |  |  |
| **Output indicators (denominators)** |  |  | |  | | |
| % of high-risk admissions correctly flagged (Number of women who were high risk i.e. risk_any) | Case note abstraction | 709 | | 1603 | | |
| % of admissions where essential information was documented in partograph and attached to case notes (total no. of observations) | Case note abstraction | 1125 | | 2034 | | |
| % of admissions where safe childbirth checklist was used and attached to case notes (total no. of observations) | Case note abstraction | 1125 | | 2034 | | |
| % of vaginal examinations where hygiene standards are met (Number of women who were examined through PV) | Observations | 142 | | 272 | | |
| % of deliveries where the six cleans were adhered to (total no. of observations) | Observations | 234 | | 392 | | |
| % of all induced deliveries where use of oxytocin protocol was indicated on case notes | Case note abstraction | Insufficient power to measure indicator at baseline. Data collection not repeated at endline | | | |  |
| % of high-risk deliveries where personnel trained in resuscitation were present | Observation of delivery |  |  |  |  |  |
| % of asphyxiated babies for which resuscitation was initiated within 1 minute | Observations of delivery |  |  |  |  |  |
| % of mothers with risk of sepsis where antibiotics were given | Case note abstraction | Not assessed at baseline _ because of very poor documentation of risk of sepsis such as fever, foul smelling discharge etc. | | | |  |
| % of babies seen in the neonatal care admission ward for whom temperature was measured within 15 minutes (total no. of observations) | Observations of admissions | 109 | 217 | |  |  |
| % of patient contacts where hygiene standards are met (total no. of observations) | Observations of patient contact in newborn care unit | 2499 | 4652 | |  |  |
| % of cannulations where hygiene standards are met (total no. of observations) | Observations of iv line | 188 | 202 | |  |  |
| % of babies discharged from newborn care unit who were exclusively breastfed at first interview after discharge (total no. of observations) | Telephonic Interviews with mothers after discharge | 866 | 1067 | |  |  |
| % of mothers in NCUs that reported being assisted for kangaroo mother care by a health worker or a relative* (total no. of observations) |  | 378 | 534 | |  |  |

*The indicator included in the protocol paper [% babies admitted to a newborn care unit for prematurity for whom the mother reports Kangaroo Mother Care”] could not be measured, because it was not possible to link the mother’s interview dataset which identifies ‘if the mother was assisted for KMC’ with ‘diagnosis of baby (prematurity)’ which was a variable in SNCU register extraction.
