# Supplementary material for: Effect of collaborative quality improvement on stillbirths, neonatal mortality and newborn care practices in hospitals of Telangana and Andhra Pradesh, India: evidence from a quasi-experimental mixed-methods study
Source: Implement Sci. 2021 Jan 7;16:4. doi: 10.1186/s13012-020-01058-z (PMC7788546; doi:10.1186/s13012-020-01058-z)
Supplement: Supplementary file 2 — Additional file 2: Annex 2: Post-hoc before and after comparison (intervention and comparison groups combined) [file 13012_2020_1058_MOESM2_ESM.docx]

**Annex 2 – Post-hoc before and after comparison (intervention and comparison groups combined)**

| **Indicator** | **Baseline**  **N=38** | **Endline**  **N=38** | **Difference**  **(95% CI)** | **p-value for t-test** |
| --- | --- | --- | --- | --- |
|  | **Mean (95% CI)** | |  |  |
| 1. Percentage of stillbirth of all hospital deliveries | 1.9  (1.4-2.5) | 0.7  (0.3-0.10) | -1.2  (-1.8 – - 0.7) | p=0.0001 |
| 1. Percentage of neonates dying before the age of 7-days among those admitted to the newborn care unit | 5.4  (2.3-8.5) | 0.9  (0.1-1.8) | -4.5  (-7.6 – -1.4) | p=0.0088 |
| 1. Percentage of neonates dying before the age of 28-days among those admitted to the newborn care unit | 7.7  (3.2-12.4) | 1.5  (0.3-2.8) | -6.2  (-10.3– -2.1) | p=0.0067 |
| **Outcome indicators** |  |  |  |  |
| 1. Percentage of high-risk assessments correctly flagged | 37.1  (29.4 – 44.8) | 19.7  (12.7-26.8) | -17.4  (-25.4 - -9.3) | P=0.0001 |
| 1. Percentage of admissions where essential information was documented in partograph and attached to case notes | 9.6  (1.7-17.4) | 14.7  (8.1 – 21.2) | 5.1  (-3.6-13.8) | p =0.24 |
| 1. Percentage of admissions where safe childbirth checklist used and attached to case notes | 11.1  (1.9-20.4) | 41.2  (25.7 – 56.7) | 30.0  (13.5-46.5) | p=0.0008 |
| 1. Percentage of vaginal examinations where hygiene standards are met | 25.0  (11.7 – 48.8) | 21.4  (2.4-40.4) | -3.6  (-28.8 – 21.5) | p=0.76 |
| 1. Percentage of deliveries where the six cleans were adhered to | 2.6  (0 – 7.3) | 6.9  (0.1- 13.4) | 4.4  (-3.3-12.1) | p=0.25 |
| 1. Percentage of babies seen in the neonatal care admission ward for whom temperature was measured within 15 minutes | 42.7  (17.1 – 68.3) | 30.4  (12.0 – 48.9) | -12.2  (-38.9 – 14.4) | p= 0.34 |
| 1. Percentage of patient contacts where hygiene standards are met | 6.3  (2.5 – 10.0) | 43.0  (31.1 – 54.9) | 36.7  (25.3-48.2) | p<0.001 |
| 1. Percentage of cannulations where hygiene standards are met | 8.7  (0 – 21.1) | 26.9  (10.3 – 43.3) | 18.1  (4.8 – 31.5) | P=0.0099 |
| 1. Percentage of babies discharged from newborn care unit who were exclusively breastfed at first interview after discharge | 96.9  (94.5 – 99.3) | 70.4  (59.5 – 81.3) | -26.5  ( -37.8 - - 15.2) | p=0.0004 |
| 1. Percentage of mothers in SNCU that reported being assisted for kangaroo mother care | 33.9  (20.2-47.5) | 57.8  (44.3 – 71.4) | 23.9  (3.1-44.8) | p=0.0257 |
