# Supplementary material for: Effect of collaborative quality improvement on stillbirths, neonatal mortality and newborn care practices in hospitals of Telangana and Andhra Pradesh, India: evidence from a quasi-experimental mixed-methods study
Source: Implement Sci. 2021 Jan 7;16:4. doi: 10.1186/s13012-020-01058-z (PMC7788546; doi:10.1186/s13012-020-01058-z)
Supplement: Supplementary file 3 — Additional file 3: Annex 3: Table 5 – Leadership, Table 6 – Contextual challenges for QI team mobilisation and capacity building, Table 7 - Perceptions of programme by health workers, Table 8 – mechanisms of change [file 13012_2020_1058_MOESM3_ESM.docx]

**Table 5 – Leadership**

| **Themes** | **Evidence from** | **Illustrative quotes** |
| --- | --- | --- |
| **Role of leaders** | | |
| Proactive, outcome-oriented leader models new behaviour and inspires change | CS1, CS4 | CS4_Round 1_Interview_04 Medical Superintendent  I: Changing behaviour of doctors and nurses with quality improvement activities is easy.  R: If the head of the family works, then the rest of the family works. I witnessed that. If the head of the family does extraordinary work, is sincere and honest then rest of the family also works. They will change. If we do extraordinary work, then they will also change |
| Providing practical support and supplies | CS1, CS2, CS4 | CS2_Round 1_Interview_03  I: Last statement for your comment – in this hospital my effort to improve quality is valued and supported  R: Administration department is quite friendly and they never object something or any intervention that is going to benefit babies. They are quite supportive and there is no problem in administrative sector. This may be problem in government sector. I never felt we should not do any kind of intervention. Administrators welcome these kind of initiatives |
| **Challenges with leadership** | | |
| Limited leadership skills or unable to motivate staff | CS3, CS4 | CS4_Round 1_Interview_2 Unit Manager NCU  I: Considering your present profile as a unit head how do you feel in supporting quality improvement activities?  R: It is very very essential. My participation and direction, I should propose and I should take very remedial measures in management. I am trying to do it but what happens you know, receiving should also play equally responsible. They don’t respond.  CS3_Round 1_Interview 09 Mentor  I: Anything else your want to tell us or any other important thing you want to share?  R: If I view from my part I will say that major thing is leadership. Some little work is going on because of XXX *(HOD labour room).* She shouts and she will tell because they won’t listen. |
| Not interested or  not sufficiently engaged | CS1, CS3, CS4 | CS4_Round 1_Interview_08 Mentor  I: Staff told us that although they all all working for QI there is no team. What do you think are the challenges in making a team?  R: In forming a team the challenges we face are XXX4 is the leader… [*does*] she knows what to do? Names of nurses… we can write as many names as you want [they mean to form a QI team] but there is a saying that root should be strong so we concentrated on the root. It is in other facility also. If one person is there to look… then they will ask the staff. So what we are doing […] how you can improve it. You make a team and make branches but if the root is not strong then…. |
| **Contextual challenges** | | |
| Professional hierarchies and boundaries affect perceptions of leadership role | CS1, CS2, CS3, CS4 | CS1_Round 1_Interview_07 Unit In-charge NCU  I: Now I will read a few statements related to quality improvement. Quality improvement works better in certain hospitals than others.  R: Quality improvement is leader’s role. Leader is in charge doctor and in charge sister both. So what I feel to manage NCU, in nursing care, sister’s role is more than doctors’ role.  CS1_Round 2_Interview_10 Medical Officer NCU  I: How do you feel about leadership?  R: Leadership is there. They (nurses) don’t consider us as a leader. They will come and do their work here and leave. |
| Top down management style prevents bottom up initiative | CS1, CS2, CS3, CS4 | CS1_Round 1_Interview_08 Medical Officer Labour Room  I: Next statement is: in this hospital my efforts to improve quality are valued and supported.  R: As an obstetrician… new initiatives if are to be followed… we have to change behavior of doctors and nurses. We *(she means junior obstetricians and senior residents)* don’t think about it; as junior subordinates we don’t give any suggestions. It will be good if they *(seniors)* will take suggestions from us… it will be good for patients. |
| Leaders do not have higher level pressure to improve quality | CS1, CS3 | CS1_R1_Interview_09 Mentor  I: I have been hearing this a lot that initially there was a lot of resistance, what was it like in xxx (this hospital?)  R: Generally there will be a resistance because […] quality is not compulsion to any Government hospital and it is their choice to implement it or not. If the leadership wants it strongly then the staff obviously do it… but they do it forcibly. If the staff wants to develop their own unit, they do it. |

**Table 6 – Contextual challenges for QI team mobilisation and capacity building**

| **Theme** | **Evidence from:** | **Illustrative quotes** |
| --- | --- | --- |
| Staff shortages and high workload prevent active engagement and result in low motivation | CS1, CS2, CS3, CS4 | CS1_Round 1_Interview 9 Mentor  I: You just mentioned that there is some problem in staffing pattern, can you explain a bit more about it… what is the issue?  R: Annual delivery numbers are 18000, so almost per month 1700, 1600 and per day it’s almost 50 to 60 deliveries are happening there. So staff present… nurses are very less, I could rarely see the nurses and when it comes to PGs, PGs […] are already overburdened and PGs shift also depends. Sometimes they work whole the day round the clock. They are only frustrated so… we couldn’t go along with them.  CS3_Round 1_Interview 4 Staff Nurse NCU  I: Did she (mentor) discuss anything about improvement?  R: No she did not because there is no staff. […] Most of us are busy, whenever she visited us. You may have noticed it too. Actually for this work four staff are required. But only two staff is working. One will be looking after all the work outside the NCU and OPD. One sister has to look after 20 babies. It’s very difficult. |
| Inadequate resources (other than staff) | CS1, CS2, CS3, CS4 | CS4_Round 1_Inteview 2 Unit Manager NCU  I: How do you feel that they are giving support on hand hygiene?  R: That is a wonderful suggestion, probably it will go long way in reducing sepsis. We are very ignorant of small things. Certainly, if they keep coming frequently for example there is no proper place for hand wash. What they tell about hand washing is true and get into our mind. But implementation? Have you seen that small hand wash basin? What possible? Nothing is possible. |
| Staff turnover, preventing continuity of engagement, particularly in labour rooms | CS1, CS2, CS3, CS4 | CS4_Round 1_Interview 5 Medical Officer labour room  I: Only few staff participate actively in the quality improvement activities.  R: Yes!  I: Why do you feel so?  R: It will be rotation always in the labour room. Staff will change, permanent staff won’t be there. Only few staffs are permanent. Juniors will come may be they are not aware. |
| Resistance from staff due to low motivation, or limited focus on quality and outcomes | CS1, CS3, CS4 | CS1_Round 1_Interview 9 Mentor  I: Only few staff participate actively in the quality improvement activities.  R: Not everyone works to improve… like some work for salaries and some work for common need and some work for job satisfaction. People who work for the job satisfaction will completely focus on what can be done next… Not everyone will have that vision. People who have that vision utilize when they have that opportunity.  CS2_Round 1_Interview 1 Medical Officer NCU  I: Quality improvement works better in certain hospitals than others.  R: Common thing which can differentiate with other hospital in quality… apart from the resources, they have to have… their thoughts to treat things… every time they think about the quality!!! Or how they can give better quality!!! and also about outcome.  CS4_Round 1_Interview 7 Unit manager Labour room  I: What can be done to change nurses’ behavious?  R: It is difficult nobody wants to work. We take salaries and we don’t work. That is attitude of the people. Every sister wants to sit daily. |

**Table 7 - Perceptions of programme by health workers**

| **Themes** | **Evidence from** | ***Illustrative quote*** |
| --- | --- | --- |
| Positive encouragement for change | CS3, CS4 | CS3_Round 2_Interview 2 Unit Manager labour room  I: If you think back about when they used to come, what was useful about their support?  R: She is a guide for us. She didn’t say it as a comment like that, she says these are the things to be done for improving the quality of services… not like somewhat criticising or pointing out like that. She is like a guide, helper for us.  CS4_Round 2_Interview 9 Staff Nurse NCU  I: How do you feel about leadership in the ward, now that xxx (mentor) is not available?  R: Yes there is a change. When she was there she used to tell us what to do and what can be done. She used to concentrate on each and every aspect and tell about checklists, monitor us, support us, tell us what new can be done, she used to encourage us. Now our work only is very heavy and we don’t have time to clearly look into all aspects so we are only filling checklists. |
| Not aware of the intervention | CS1, CS2, CS3, CS4 | CS3_Round 1_Interview 06 Medical officer NCU  I: I am asking about the ACCESS team, they work as mentor and visit the unit frequently on quality improvement activities?  R: I think I saw once ACCESS people that’s it. I haven’t interacted. Actually they asked me something about the new born care unit. I asked them to please provide the details of the program, department details and from where they are coming, about your project but they denied. They haven’t got that details.  CS4_Round 1_Interview 02 Unit Manager NCU  I: Did they share the data regarding what is going on NCU?  R: They come and sit here and open their laptop, then they discuss with madam (medical officer) at length and go away. The data they have not shared. […]  I2: As unit manager, can you tell what Access can support in your unit in quality improvement?  R: I don’t know what ACCESS does but what they told us initially was regarding hand washing |
| Programme perceived as an external assessment | CS1, CS3, CS4 | CS1_Round 1_Interview 04_ Staff Nurse NCU  I: You just mentioned that they assess. What kind of assessment is this?  R: They assess “whether we are practicing hand wash or using hand rub”. They observe us and if we are free, they come and also ask us.  I: What they do with assessment?  R: I think they tell unit-manager and medical officers  CS3_Round 2_Interview 02 Labour room in-charge  I: Will they come back and share the …?  R: They will come back and see what have been done as advised by them. Some are done and some have not been done. |
| Programme increases workload related to documentation | CS1, CS3, CS4 | CS1_Round 2_Interview 11 Staff Nurse NCU  I: Why has the use of the checklist stopped?  R: We are busy and there is nobody to ask about it. We monitor but not document. We guide each other orally.  CS4_Round 2_Interview 09 Staff Nurse NCU  I: With the data collected, are you preparing any graph?  R: No, I am not preparing any graph. We don’t have enough time for that activity.  I: Do you know how to prepare the graph?  R: Yes, we know how to prepare the graph, but we are not doing it. |
| Programme has limited acceptability due to limited clinical offer | CS2, CS4 | CS2_Round 1_Interview 5 Mentor  I: How does the hospital perceive you?  R: If you know that how corticosteroids administration impact in to pre-maturity? If you correlate that aspect … basically the knowledge aspect… the respect is inevitable. […]age that doesn’t mean… but some twenty-year [old] guy goes and directs the doctor… is he going to give same response as when a 40 year old guy commanding a doctor, they are not going to listen. So the way you present the work and the way you support them. That is very important apart from the age.  CS4_Round 1_Interview 8 Mentor  I: Is there anything that you feel you or SCSL could have done differently?  R: It requires time. We were in need of paediatricians, gynaecologists, apart from programme staff going to the facility. That could have added much more for the programme. |
| Programme does not offer relative advantage, or is not appropriate | CS1, CS2, CS3, CS4 | CS4_Round 1_ Interview 7 Unit Manager Labour room  I: How do you feel abut the support provided by the ACCESS Health?  R: I feel support is not appropriate. The Government or non-governmental organization work in a different level and everyone wants to work at above the ground level. Thereby you don’t understand the things what goes at ground level. Nobody is trying to educate people at village level.  CS4_Round 1_Interview 3 Staff Nurse NCU  I - How did ACCESS provide support to you?  R - When they come they speak to mam only. They don’t speak to us. They tell to prevent sepsis, do hand wash. They speak in Hindi and we don’t know. So they speak to mam they don’t speak much to us. |

**Table 8 – mechanisms of change**

| **Themes** | **Evidence from** | **Illustrative quote** |
| --- | --- | --- |
| Intervention brings new focus and new ideas | CS1, CS2, CS3, CS4 | CS1_Round 1_Interview 4 Staff Nurse NCU  I: How did it happen? [referring to more staff practicing handwashing now]  R: Before no one used to focus on these things. Once they came, Madam started insisting on these things. We didn’t like it before, but later on we thought what they say is true, we should follow this.  CS4_Round 1_Interview 1 Medical Officer NCU  I: All these changes are happening now, why they were not happening before? What is your opinion?  R: Previously I was not aware of all these things. We were FBNC trained [*trained in Facility Based Newborn Care*]. We were not so cautious and not so focused. After giving the targets, not every case, at least on three areas where we have to focus, we are planning to focus on these three and improve for their outcomes. |
| Intervention improves motivation and commitment to the aim | CS1, CS2, CS3, CS4 | CS3_Round 2_Interview 4  I: Did you know before ACCESS came here, about the advantage of handwashing?  R: Yes we know it.  I: Why did you start after ACCESS has come here, why you did not do it before?  R: They told that sepsis can be controlled and we can send the babies home faster. So by doing few changes we also felt good. |
| Intervention fills skills gaps, reinforces knowledge and provides opportunity to learn something new | CS1,CS2, CS3, CS4 | CS1_R1_07 Unit In charge NCU  I: How is ACCESS working with you for quality improvement?  R: They are helping us in improving our hand washing techniques, and putting intracaths, these two techniques they have improved. Actually I tried my level best to change the attitude of our staff to improve hand washing practices. But it was difficult. And these ACCESS health people they are conducting trainings, they are giving health education to our staff, after that there was change in hand hygiene including scrubbing in between touching babies and everything was improved. There was dramatic improvement. |
| Improved sense of personal responsibility | CS1, CS3, CS4 | CS1_Round 1_Interview 5 Medical Officer NCU  Anything before advising others, we have to practice ourselves first or else there will not be any value for that. This I believe strongly… see before I advise to nurses, I should be able to do or know to do that practice. If I don’t follow the practice, staff may say that we don’t follow ourselves but ask others to perform the procedure. So this made me more conscious about myself.  CS4_Round 1_Interview 1 Medical Officer NCU  I: What are the steps you have taken to come out of that negative result?  R: First thing I am planning to put 100 percent effort from myself individually. I have to be strict from my side. I should wear glove whenever I touch the baby like. I am planning to correct myself then I am trying to help the staff nurses. […]  From my side I see change in myself also. Because it was like I was *bindaas* (means careless) in touching all the babies nicely. Now I am like, ok, now I should not touch the baby without hand rub at least. I am cautious from my side in maintaining asepsis especially. |
| New standards established | CS1, CS3, Cs4 | CS4_Round 2_Interview 9 Staff Nurse NCU  R – They will do compulsorily. Previously they used to not do that. But now after the quality improvement people have come they do compulsorily hand wash and use hand rub in between. If they forget also, we remind them. They don’t feel [bad] because we are seniors they know why we are saying. Now everyone is aware that they should do hands wash. |
| **Challenges** |  |  |
| Continuous scrutiny is needed | CS1, CS3, CS4 | CS3_Round 1_Interview 4 Staff Nurse NCU  I: What other things are lacking?  R: Lack of motivation. Unless and until we are at the back of them and encourage them to do, they will not perform. If we tell one time they may not be able to do it. Now you are coming. If you give us some work and ask us to do, we will perform that activity only if we know that you are going to come back tomorrow to verify the same. If you come once in a blue moon day and ask us to do something, then they will not do it. The staff needs to have fear that people are coming back to ask us again.  CS4_Round 1_Interview 4 Medical Superintendent  I: Sir, what else do you think is necessary to achieve and do to improve quality?  R: Uninformed frequent and random visits are required. The visits should be totally uninformed. Then they can access what we are doing? If these type of actions are undertaken, 100 percent we can see improvement. |
| Mindset change but only in few individuals | CS1, CS2, CS3, CS4 | CS1_Round 1_Interview 4 Staff Nurse NCU  R: Yes Ma’am, I told them. Everyone was telling me that one assistant is required. After putting cannulation, securing it is difficult. I showed them how is it possible to do single-handedly when somebody is not available. I showed them it is possible to do by one person.  I: How did they react when you showed this to them?  R: They said only you can do this. We can’t. *[Respondent laughing]*  I: Later did they try to learn?  R: No Ma’am  CS1_Round 1_Interview 5 Medical Officer NCU  I: Why do you feel that some staff do not participate actively in the team? What could be the reason?  R: Few staff’s mindset is… like to come to work simply and go off. They are doing their work but it’s kind of mechanical work. May be that mindset has to change. This is my direct feeling and I just can’t hide what I feel. |
